# ACE Inhibitors Boost Mobility and Muscle Strength by Reducing Intestinal Permeability in Older Adults with Alzheimer’s Disease

**DOI:** 10.3390/ph19020304

**Published:** 2026-02-12

**Authors:** Rizwan Qaisar, Asima Karim, M. Shahid Iqbal, Firdos Ahmad, Khalid Saeed, Shaea A. Alkahtani

**Affiliations:** 1 Basic Medical Sciences, College of Medicine, University of Sharjah, Sharjah 27272, United Arab Emirates; rqaisar@sharjah.ac.ae (R.Q.); akarim@sharjah.ac.ae (A.K.); fahmad@sharjah.ac.ae (F.A.); 2 Space Medicine Research Group, Research Institute of Medical and Health Sciences, University of Sharjah, Sharjah 27272, United Arab Emirates; 3 Cardiovascular Research Group, Research Institute of Medical and Health Sciences, University of Sharjah, Sharjah 27272, United Arab Emirates; 4 Department of Neurology and Stroke Medicine, Rehman Medical Institute, Peshawar 25120, Pakistan; shahid.iqbal@rmi.edu.pk; 5 Drug Discovery Group, University of Helsinki, 00170 Helsinki, Finland; khalid.saeed@helsinki.fi; 6 Exercise Physiology Department, College of Sport Sciences and Physical Activity, King Saud University, Riyadh 11451, Saudi Arabia

**Keywords:** Alzheimer’s disease, hypertension, handgrip strength, zonulin, ACE inhibitors, SPPB

## Abstract

**Objectives:** Hypertension is common in Alzheimer’s disease (AD) and contributes to functional decline. While ACE inhibitors are widely used for hypertension, their systemic effects on intestinal permeability and physical capacity in AD patients remain unclear. **Materials and Methods:** We investigated the potential contribution of increased intestinal permeability to handgrip strength (HGS) and physical capacity in patients with Alzheimer’s disease (AD) taking ACE inhibitors. We investigated hypertensive AD patients taking ACE inhibitors (*n* = 55) or other anti-hypertensive medications (*n* = 57) at baseline and one year later, along with age-matched controls (*n* = 64) and normotensive AD patients (*n* = 61). We measured plasma zonulin, a marker of intestinal permeability, and HGS, and performed the short physical performance battery (SPPB). **Results:** AD patients had lower HGS, gait speed, SPPB, and higher plasma zonulin than controls at baseline (all *p* < 0.05). The use of ACE inhibitors was associated with increased HGS and gait speed, and reduced plasma zonulin in AD patients. Conversely, AD patients on other anti-hypertensive medications had higher zonulin and lower HGS but no change in gait speed and SPPB after one year. The patients taking ACE inhibitors also exhibited significant dynamic correlations of zonulin with HGS, gait speed, and SPPB (*p* < 0.05). ACE inhibitors also reduced plasma C-reactive proteins and 8-isoprostanes as markers of oxidative stress and inflammation. **Conclusions:** ACE inhibitors may improve physical performance and cognitive function in hypertensive AD patients, primarily through vascular smooth muscle modulation, leading to better perfusion. These effects may indirectly support intestinal barrier and muscle function, highlighting a novel gut–vascular–muscle interface relevant to therapeutic strategies.

## 1. Introduction

Hypertension is a common finding in patients with advanced old age and is also an independent risk factor for developing Alzheimer’s disease (AD) [1]. It contributes to vascular dysfunction, oxidative stress, inflammation, and reduced cerebral blood flow in patients with chronic hypertension [2]. Patients with AD are also more likely to develop an early and severe form of age-related muscle decline, termed sarcopenia [3]. Specifically, these patients exhibit muscle weakness, atrophy, and reduced gait speed, which can contribute to compromised physical capacity [3]. Sarcopenic patients are also more likely to have a physically dependent lifestyle and a higher risk of falls than the age-matched controls [3].

Sarcopenia has a multifactorial etiology, and it is possible that a progressive denervation of skeletal muscle in AD patients contributes to sarcopenia [3,4]. In addition, long-term systemic hypertension may also negatively affect skeletal muscle [5]. Chronic hypertension can cause vascular damage and reduced blood flow to skeletal muscles, leading to ischemia and subsequent muscle impairment. Additionally, hypertension is associated with increased oxidative stress and inflammation, which can further damage skeletal muscle. Lastly, hypertension activates muscle catabolic pathways, such as the ubiquitin-proteasome system, leading to increased protein degradation and muscle loss [5,6]. Together, these factors may account for a higher occurrence of sarcopenia in AD patients compared to age-matched controls.

Despite a lack of direct pathology, AD patients experience a range of systemic manifestations [7]. Among those, a pathologically increased intestinal permeability may be particularly important [8]. Intestinal permeability is primarily regulated by epithelial tight junctions, smooth muscle tone, and microvascular function. Zonulin plays a key role in modulating tight junction integrity, making it a relevant biomarker in this study. This alteration impacts multiple body organs, notably skeletal muscle. For example, recent research has highlighted the role of increased intestinal permeability in the development of sarcopenia [9]. Intestinal permeability can be assessed through several circulating biomarkers, including intestinal fatty acid binding protein, zonulin, and lipopolysaccharide binding protein [10]. The regulatory effects of zonulin on the structure and function of intestinal mucosal tight junctions make it particularly relevant in this context [11]. The patients with advanced sarcopenia exhibit higher levels of plasma zonulin [3,9]. Plasma zonulin is an acute-phase reactant and can contribute to heightened intestinal permeability by loosening the tight junctions in the gut lining [11]. The resultant intestinal leak allows harmful substances, including bacteria and their products, to enter the bloodstream, triggering systemic inflammation and oxidative stress [12]. In addition, a leaky gut also reduces the nutrients required for muscle maintenance [12]. Collectively, these factors negatively impact skeletal muscle by promoting muscle protein breakdown and inhibiting muscle protein synthesis, ultimately contributing to the sarcopenia process. Further, plasma zonulin levels also correlate with the degree of dementia, indicating a potential causal relationship between increased intestinal permeability and cognitive decline [3].

The increased intestinal permeability in AD may partly be due to systemic hypertension. In support of this, hypertension is associated with intestinal mucosal disruption, intestinal dysbiosis, and increased inflammatory status [13]. In addition, plasma zonulin levels are higher in hypertensive vs. normotensive patients and exhibit a direct correlation with systolic blood pressure [14]. These detrimental effects of intestinal disruption are partly due to the translocation of harmful bacteria and their metabolites into circulation. Among them, the lipopolysaccharides and short-chain fatty acids cause an increased oxidative stress and inflammation in the target tissues [12]. In addition, elevated systemic inflammation is also commonly associated with intestinal disruption [12]. The detrimental effects of oxidative stress and inflammation on skeletal muscle are well established [4,15]. Together, these findings indicate that hypertension-associated intestinal mucosal disruption may contribute to skeletal muscle decline and reduced physical capacity in AD patients.

Because hypertension is associated with increased intestinal permeability, anti-hypertensive medications could potentially reduce intestinal permeability and its associated systemic adverse effects. These drugs work through various mechanisms and have different levels of impact. Angiotensin-converting enzyme (ACE) inhibitors are of particular interest, as recent research indicates their protective effects against dementia and skeletal muscle decline, possibly mediated in part by blood pressure reduction [16,17]. ACE inhibitors act by inhibiting angiotensin II, reducing vascular smooth muscle tone, and improving endothelial function. These changes enhance cerebral and peripheral blood flow, which may explain their protective effects on cognition and physical capacity. Animal studies also indicate that ACE inhibitors are associated with a reversal of intestinal permeability and fibrosis, and hypertrophy of intestinal smooth muscles [18,19]. Thus, it is possible that the potential protective effects of ACE inhibitors against dementia and sarcopenia are at least partly due to the strengthening of the intestinal mucosal barrier. However, similar effects of ACE inhibitors in clinical settings in the context of AD and sarcopenia are not known.

We have previously reported that ACE inhibitors preserve the neuromuscular junction in skeletal muscle, which may contribute to improved muscle strength and physical performance [20]. While NMJs are not directly relevant to hypertension or vascular physiology, these findings highlight the broader systemic benefits of ACE inhibitors. However, the relevant effects of ACE inhibitors on intestinal permeability and the subsequent influence on dementia and physical capacity are unknown.

We aimed to investigate the associations of intestinal permeability with dementia and physical capacity in hypertensive AD patients taking ACE inhibitors. Plasma zonulin levels were measured as a marker of intestinal permeability. We hypothesized that ACE inhibitors repair intestinal leaks and reduce plasma zonulin, which, in turn, is associated with improved cognition and physical capacity in AD patients.

## 2. Results

### 2.1. Characteristics of the Participants

Table 1 describes the basic characteristics of controls (age = 74.6 ± 4.5 years; *n* = 64) and AD patients. The AD patients were categorized into normotensive (age = 76.3 ± 3.8 years; *n* = 61) and two groups of hypertensive patients receiving ACE inhibitors (age = 74.9 ± 4.2 years; *n* = 55) or other anti-hypertensive medications (age = 76.1 ± 4.7 years; *n* = 57). We found no change in BMI, ASM, and percentage fat in AD patients for a one-year period. All AD patients exhibited lower phase angles than controls (all *p* < 0.05) (Table 1). Among three groups of AD patients, only the patients on ACE inhibitors exhibited a significant improvement in phase angle (*p* < 0.05). The AD patients also showed higher creatine kinase and lower MMSE scores than controls, which were not affected during the study period. We also found a trend towards a higher prevalence of sarcopenia among AD patients, which was not affected over a one-year period.

### 2.2. Plasma Biomarkers and Sarcopenia Parameters

**Figure 1:**
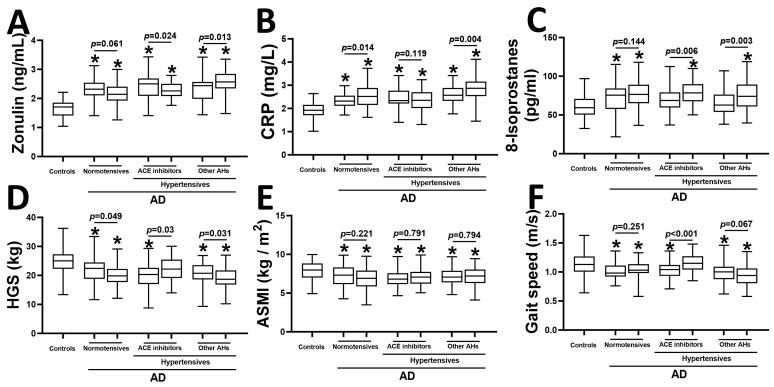
Plasma levels of zonulin (**A**), CRP (**B**), 8-isoprostanes (**C**), and HGS (**D**), ASMI (**E**), and gait speed (**F**) in controls, normotensive, and hypertensive AD patients taking ACE inhibitors or other anti-hypertensive medications. * *p* < 0.05 vs. controls using one-way analysis of variance. *p*-values indicate paired *t*-tests in the same group of patients at two timepoints, one year apart (*n* = 55–64 per group). CRP; c-reactive protein, HGS; handgrip strength, ASMI; appendicular skeletal mass index, ACE; angiotensin-converting enzymes, AD; Alzheimer’s disease.

We next investigated the plasma levels of zonulin, CRP (a marker of inflammation), and 8-isoprostanes (a marker of oxidative stress), as well as sarcopenia parameters, including HGS, ASMI, and gait speed, in the study population (Figure 1). All AD patients exhibited higher plasma zonulin than controls (all *p* < 0.05) (Figure 1A). Patients taking ACE inhibitors had a significant reduction in plasma zonulin after one year (*p* = 0.024). Separately, the use of other anti-hypertensive medications was associated with an elevation of plasma zonulin (*p* = 0.013). AD was also associated with elevated CRP than controls (all *p* < 0.05) (Figure 1B). There was further elevation of plasma CRP in normotensive AD patients (*p* = 0.014) and hypertensive patients taking other anti-hypertensive medications (*p* = 0.004) after one year (Figure 1B). We also observed an increase in plasma 8-isoprostane levels in AD patients taking ACE inhibitors (*p* = 0.006) or other anti-hypertensive medications (*p* = 0.003) (Figure 1C).

Among sarcopenia parameters, HGS was significantly lower in all AD patients than in controls at baseline (all *p* < 0.05) (Figure 1D). After one year, we observed a reduction in HGS in normotensive AD patients (*p* = 0.049) and patients taking other anti-hypertensive medications (*p* = 0.031). Additionally, the use of ACE inhibitors was associated with an increase in HGS in AD patients (*p* = 0.03) (Figure 1D). We also found lower ASMI in all AD patients compared to controls (all *p* < 0.05), which remained unchanged during the one-year study period (Figure 1E). Lastly, all AD patients exhibited lower gait speed than controls (all *p* < 0.05) (Figure 1F). Among the three groups of AD patients, only the hypertensive patients taking ACE inhibitors exhibited an improvement in gait speed after one year (*p* < 0.001) (Figure 1F).

### 2.3. SPPB Scores and Their Associations with Plasma Zonulin

**Figure 2:**
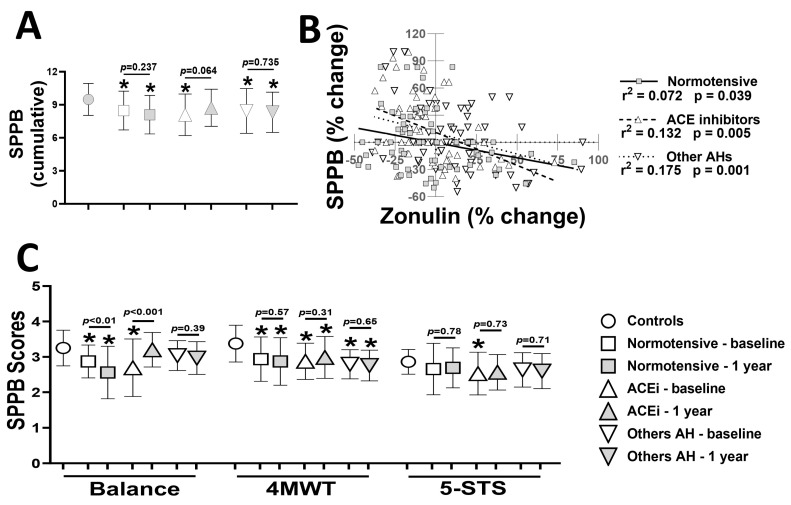
Cumulative SPPB scores (**A**), linear regression analysis of associations of alterations in SPPB with zonulin (**B**), and the scores for balance, 4MWT, and 5-STS components of SPPB (**C**) in controls, normotensive, and hypertensive AD patients taking ACE inhibitors or other anti-hypertensive medications. * *p* < 0.05 vs. controls using one-way analysis of variance. *p*-values indicate paired *t*-tests in the same group of patients at two timepoints, one year apart (*n* = 55–64 per group). SPPB; Short physical performance battery, 4MWT; 4—meter walking test, 5—STS; five times chair sit-to-stand test, ACE; angiotensin-converting enzymes.

We next investigated SPPB scores as markers of physical capacity in controls and AD patients (Figure 2). At baseline, the cumulative SPPB scores were lower in all AD patients than in controls (all *p* < 0.05) (Figure 2A). The three groups of AD patients exhibited no change in SPPB scores after one year, despite a trend towards higher scores in patients taking ACE inhibitors (*p* = 0.064). We next investigated the associations between alterations in SPPB and plasma zonulin for a one-year period in AD patients (Figure 2B). All three groups of AD patients exhibited significant associations between SPPB and plasma zonulin, which were more robust for hypertensive patients taking ACE inhibitors (r^2^ = 0.132, *p* = 0.005) or other anti-hypertensive medications (r^2^ = 0.175, *p* = 0.001) (Figure 2B). Among the individual SPPB components, the performance on 5-STS was generally poorer than the other two components (Figure 2C). The use of ACE inhibitors was associated with a significant improvement in maintaining balance in AD patients (*p* < 0.001). Lastly, other anti-hypertensive medications had no effects on the individual SPPB components (Figure 2C).

### 2.4. The Dynamic Associations of Plasma Zonulin with Sarcopenia Parameters

**Figure 3:**
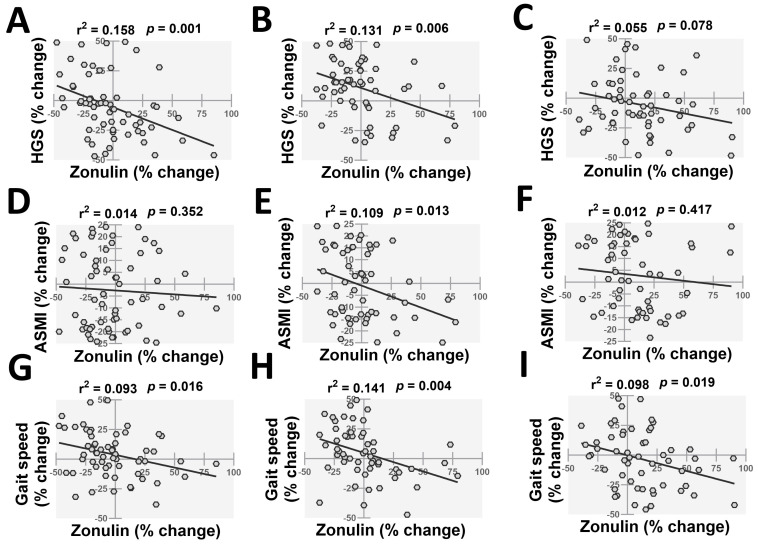
Linear regression analysis showing the associations of alterations in HGS (**A**–**C**), ASMI (**D**–**F**), and gait speed (**G**–**I**) with plasma zonulin levels in normotensive (**A**,**D**,**G**) and hypertensive AD patients taking ACE inhibitors (**B**,**E**,**H**) or other anti-hypertensive (**C**,**F**,**I**) medications during one year of study period (*n* = 55–64 per group). HGS; handgrip strength, ASMI; appendicular skeletal mass index.

We next investigated the associations of changes in plasma zonulin with sarcopenia parameters in the three groups of AD patients (Figure 3). HGS exhibited significant associations with plasma zonulin in normotensive (r^2^ = 0.158, *p* = 0.001) (Figure 3A) and hypertensive patients taking ACE inhibitors (r^2^ = 0.131, *p* = 0.006) (Figure 3B). However, the association between HGS and zonulin was not significant in hypertensive AD patients taking other anti-hypertensive medications (r^2^ = 0.055, *p* = 0.078) (Figure 3C). We next investigated the associations between ASMI and plasma zonulin in AD patients (Figure 3D–F). Only patients taking ACE inhibitors demonstrated a significant association between ASMI and plasma zonulin (r^2^ = 0.109, *p* = 0.013) (Figure 3E). Lastly, gait speed exhibited significant associations with plasma zonulin in normotensive (r^2^ = 0.093, *p* = 0.016) (Figure 3G) and hypertensive AD patients taking ACE inhibitors (r^2^ = 0.141, *p* = 0.004) (Figure 3H) or other anti-hypertensive medications (r^2^ = 0.098, *p* = 0.019) (Figure 3I).

### 2.5. Associations of Plasma Zonulin with MMSE, Sarcopenia Parameters, Physical Capacity, and Plasma Biochemistry

We last investigated the associations of plasma zonulin with different parameters at individual timepoints (Table 2). MMSE scores exhibited a significant association with plasma zonulin only in AD patients taking ACE inhibitors (r^2^ = 0.123, *p* < 0.05). We found varying degrees of associations of plasma zonulin with various sarcopenia parameters and SPPB scores. AD patients taking ACE inhibitors at baseline exhibited the most robust associations of plasma zonulin with HGS (r^2^ = 0.214, *p* < 0.05) and gait speed (r^2^ = 0.187, *p* < 0.05) (Table 2). Among plasma biochemical markers, CRP exhibited the most consistent and robust associations with zonulin in different groups of AD patients. Conversely, the associations of plasma CK and 8-isoprostanes with zonulin were relatively less robust (Table 2).

## 3. Discussion

The patients with AD exhibited reduced HGS and physical capacity along with elevated plasma zonulin levels. The use of ACE inhibitors was associated with an improvement in HGS and a reduction in plasma zonulin. However, ACE inhibitors were not associated with improved SPPB scores despite a trend towards an increase. These effects of ACE inhibitors were at least partly mediated by a reduction in plasma zonulin, as evidenced by robust dynamic correlations between alterations in plasma zonulin and HGS, gait speed, and SPPB.

Intestinal permeability is governed by the integrity of epithelial tight junctions, smooth muscle tone, and microvascular function. Our findings suggest that ACE inhibitors may indirectly support these mechanisms by reducing systemic inflammation and oxidative stress. NMJs are unrelated to intestinal permeability and were not examined in this study.

Plasma zonulin was used in this study as a surrogate marker of intestinal permeability, but its limitations must be acknowledged. Zonulin reflects the functional regulation of epithelial tight junctions rather than direct paracellular flux across the intestinal barrier, and circulating levels are influenced by inflammatory and metabolic signals that affect biological specificity [11]. In addition, currently available commercial assays may detect zonulin-related proteins rather than pre-haptoglobin-2 itself, a recognized limitation in zonulin-based studies [21]. Therefore, the associations reported here should be interpreted as indicators of altered tight junction regulation and gut barrier signaling rather than as definitive evidence of structural intestinal leakiness. Alternative permeability assessment tools include the lactulose–mannitol test, intestinal fatty acid-binding protein, and lipopolysaccharide-binding protein [10]. However, lactulose–mannitol testing requires strict dietary control and urine collection, which is challenging in older adults with cognitive impairment, while intestinal fatty acid binding protein reflects acute enterocyte injury rather than chronic barrier regulation, and lipopolysaccharide binding protein is strongly influenced by hepatic clearance and systemic inflammation [10]. For these reasons, zonulin was selected as the most feasible and clinically applicable marker for longitudinal assessment in patients with Alzheimer’s disease.

Previous studies show that ACE inhibitors were associated with reduced sarcopenia phenotype and improved physical performance in older human adults [17,22]. However, the relevance of plasma zonulin to ACE inhibitors and physical capacity in clinical settings was poorly known. On the other hand, rats with chronic hypertension display a reduction in intestinal permeability and dysbiosis following the infusion of ACE inhibitors [13]. These findings are consistent with reduced zonulin levels observed in our cohort. Interestingly, the animal models exhibit sustained offsetting effects of ACE inhibitors on hypertension, intestinal dysbiosis, and mucosal disruption after the treatment is discontinued [23]. The sustained effects of ACE inhibitors are attributed to their long-lasting impact on the gut–brain axis.

In line with previous studies, we found that the use of ACE inhibitors was associated with several protective effects on skeletal muscle, including improved HGS [17]. These effects, at least in part, contributed to improved physical capacity and gait speed. Several potential mechanisms are suggested to explain the protective effects of ACE inhibitors on skeletal muscle [17]. Our findings suggest that ACE inhibitors may improve physical capacity through mechanisms beyond blood pressure control, including modulation of intestinal permeability. Previous work on NMJs in skeletal muscle supports the idea that ACE inhibitors exert systemic benefits, although NMJs were not examined in this study. While this conclusion is speculative, several indirect evidence support it. For example, intestinal dysbiosis and mucosal disruption have been previously shown to contribute to muscle weakness and physical compromise in older adults [24]. This is at least partly due to the translocation and leakage of harmful bacterial products from the intestinal lumen to the systemic circulation [12]. These products include lipopolysaccharides and several pro-inflammatory cytokines, which together increase the oxidative stress and inflammation [12]. Previous research has critically established the susceptibility of skeletal muscle to oxidative stress and inflammation [4,15]. For example, oxidative stress disrupts several cellular pathways associated with excitation-contraction coupling and protein synthesis, which leads to muscle weakness and atrophy. Similarly, systemic inflammatory conditions, such as COPD, also led to a reduction in muscle strength and mass [12]. Thus, the elevated plasma 8-isoprostane and CRP levels may partly explain the mechanistic interface between intestinal mucosal disruption and muscle decline in AD patients. However, ACE inhibitors also have direct antioxidant and anti-inflammatory properties. For example, three months of treatment with captopril reduces glutathione levels and systemic markers of lipid peroxidation in diabetic patients [25]. ACE inhibitors also reduce pro-inflammatory cytokines and promote anti-inflammatory cytokines in old age, which together reduce the frailty phenotype and improve skeletal muscle [26]. Further, the protective effects of ACE inhibitors on muscle strength and gait speed should be interpreted as downstream effects of enhanced vascular function rather than direct skeletal muscle protection. Improved perfusion and reduced oxidative stress likely contribute to better neuromuscular performance.

Contrary to an improvement in HGS and gait speed, the cumulative SPPB scores did not improve in patients taking ACE inhibitors. However, we found an improvement in maintaining balance following the administration of ACE inhibitors. These findings indicate that the improved muscle function in patients with ACE inhibitors contributes to the maintenance of postural balance in AD patients. The postural stability is governed by several body systems beyond skeletal muscle, including the motor nervous system, the cardiovascular system, and the respiratory system [27,28]. ACE inhibitors exhibit protective effects against the decline of these systems [26,29,30], which may contribute to maintaining postural balance in our study cohort.

AD patients exhibited lower ASMIs than controls, which agrees with our previous findings [3]. However, we found no alterations in ASMI in all AD patients over the study period, irrespective of the medication status. Muscle mass remains relatively stable compared to muscle force-generating capacity in various disease conditions [31,32]. Thus, it is possible that the onset of muscle atrophy may be delayed when compared to muscle weakness in AD patients. Interestingly, plasma CK, a marker of muscle damage, was elevated in hypertensive AD patients taking other anti-hypertensive medications. Conversely, patients taking ACE inhibitors exhibited a trend toward a reduction in plasma CK levels.

We did not observe an increase in ASMI in patients on ACE inhibitors, which is consistent with our recent finding in patients with congestive heart failure [33]. This could be because ACE inhibitors primarily work by reducing angiotensin II, which is more associated with muscle function and strength than mass [34]. Activation of angiotensin II contributes to muscle loss and fibrosis, and its inhibition helps maintain muscle quality and function. However, this action does not necessarily trigger the anabolic pathways required for muscle growth. Furthermore, while ACE inhibitors can improve blood flow and nutrient delivery, they do not directly stimulate muscle protein synthesis for increased mass.

Other antihypertensive medications may also have the potential to strengthen the intestinal mucosal barrier and reduce plasma zonulin levels, although direct evidence for these effects is currently lacking. Beta blockers have been shown to improve intestinal permeability in patients with cirrhosis and portal hypertension, likely due to their anti-inflammatory properties [35]. This protective effect might also reduce plasma zonulin levels, suggesting enhanced tight junction integrity. Diuretics, particularly loop diuretics, can influence the expression of claudin proteins, which are essential for maintaining tight junction integrity [36]. Calcium channel blockers reduce colonic contractility and tone, which may help protect against leaks in the intestinal mucosal barrier [37]. However, their direct impact on intestinal permeability and plasma zonulin levels remains underexplored. Similarly, the relevance of these effects of other anti-hypertensive medications to muscle restoration is poorly understood.

The observed changes in physical and biochemical parameters have clinical relevance. HGS values below 27 kg in men are associated with disability and functional dependence, and small increases are linked to better clinical outcomes in older adults [38]. The improvement in HGS observed in patients receiving ACE inhibitors, therefore, suggests preservation of muscle function. Similarly, gait speed below 0.8 m/s predicts falls and loss of independent mobility, while increases of 0.05–0.10 m/s are considered clinically meaningful [39]. The magnitude of gait speed improvement seen in the ACE inhibitor group falls within this range. Elevated zonulin and inflammatory markers such as CRP are associated with frailty and physical decline, and their reduction may reflect biologically meaningful improvements in gut barrier regulation and inflammatory burden that support functional independence. However, our findings should be interpreted cautiously. Although ACE inhibitor use was associated with improved skeletal muscle and reduced intestinal permeability, these observational data do not support preferential prescribing of ACE inhibitors solely to improve physical function in hypertensive patients with AD. ACE inhibitors offer established vascular and anti-inflammatory benefits but also carry risks, including hypotension, renal dysfunction, cough, and electrolyte disturbances, which require careful consideration in older adults. Compared with other antihypertensive classes, ACE inhibitors may confer additional pleiotropic effects; however, their relative advantages for muscle and gut health remain unproven.

This study has several strengths. The study was conducted at a single center, and all participants had relatively similar lifestyles and ethnic backgrounds. Thus, we are confident that the potential variations in plasma zonulin levels due to diet, ethnicity, and genetics are minimal. The HGS measurements and ELISA assays for plasma zonulin do not require extensive technical expertise and can be performed in most clinics. However, this study has certain limitations. Due to recruitment difficulties arising from cultural factors, female participants were excluded from this study. This exclusion limits the generalization of our findings to both genders. This is because women show different sarcopenia severity and progression, as evident by different diagnostic criteria from men [40]. Women also exhibit different ACE activity, affecting their potential response to ACE inhibitors compared to men [41]. We did not measure lower limb muscle strength, which may be more pertinent to physical dependency. All participants were ambulant, so our findings may be cautiously interpreted in non-ambulant patients. The inclusion of only Caucasian men limits generalizability, as biological sex differences may influence ACE inhibitor responses and gut–muscle interactions. Women exhibit lower ACE activity due to hormonal regulation of the renin–angiotensin system, which may modify vascular and inflammatory responses to ACE inhibition [42]. In addition, AD shows sex-dependent differences in prevalence and vascular pathology, which may influence interactions between hypertension, gut barrier function, and physical decline [43]. These factors should be considered when interpreting the present findings. Another limitation of this study is the absence of longitudinal blood pressure measurements and a detailed assessment of hypertension control across treatment groups. Accordingly, it cannot be determined whether the observed improvements in zonulin, muscle strength, and gait speed in patients receiving ACE inhibitors are independent of blood pressure reduction or primarily reflect improved control of hypertension. The proposed improvement in tissue perfusion should therefore be interpreted as a combined effect of blood pressure lowering and pleiotropic actions of ACE inhibitors on vascular function rather than a blood pressure–independent mechanism [44]. Future studies incorporating longitudinal blood pressure data will be required to clarify this distinction. This study is observational in nature and therefore does not allow causal inference. The associations observed between ACE inhibitor use, intestinal permeability markers, inflammatory status, and physical performance should be interpreted as correlative rather than causal. Another limitation of this study is that the clinical rationale for prescribing ACE inhibitors versus other antihypertensive medications was not systematically captured. Antihypertensive therapy was selected by treating physicians in accordance with routine clinical practice, which may have been influenced by comorbidities, prior medication tolerance, or physician preference. The sample size of this study should also be considered when interpreting the results. With group sizes ranging from 55 to 64 participants, the study was adequately powered to detect moderate between-group differences and associations but may have been underpowered to identify small effect sizes or subtle dose–response relationships. As a result, some meaningful associations may not have reached statistical significance. Detailed information on gastrointestinal disorders, recent antibiotic or probiotic use, dietary patterns, and lifestyle factors such as physical activity and smoking was not systematically collected. These factors may independently influence intestinal permeability and circulating zonulin levels and therefore represent potential sources of residual confounding. Although all participants were ambulant and shared similar regional dietary habits, future studies should incorporate structured assessment of gut-related comorbidities and lifestyle exposures.

In conclusion, we demonstrate that the improvement in HGS and gait speed in AD patients taking ACE inhibitors may partly be due to the strengthening of the intestinal mucosal barrier. Moreover, plasma zonulin may be a useful biomarker of HGS and physical capacity in older adults with AD. Future studies should validate and expand the potential mechanistic association between intestinal permeability and physical capacity in AD patients.

## 4. Materials and Methods

### 4.1. Study Design & Participants

We recruited Caucasian men, including controls and patients with AD, for this study. All study participants were Caucasian men. We categorized AD patients into normotensive and two groups of hypertensive patients receiving ACE inhibitors or other anti-hypertensive medications. The ACE inhibitors include captopril (25–50 mg/day) and lisinopril (10–20 mg/day), which are centrally acting medications [16]. The other anti-hypertensive medications include beta-blockers, calcium channel blockers, and diuretics. All patients receiving ACE inhibitors had been on treatment for at least several months prior to baseline assessment, and no major changes in antihypertensive class occurred during the one-year follow-up period. We used a mini-mental state examination (MMSE) to diagnose AD, as described elsewhere [45]. The recruitment criteria for inclusion and exclusion of patients have been previously described in detail by us [3]. Briefly, patients with arthritis, major musculoskeletal disorders, recent major surgeries, and major organ failure were excluded. We recruited age- and gender-matched controls from a large representative cohort. Sarcopenia was diagnosed according to the revised criteria by the European Working Group on Sarcopenia in Older People, as reduced handgrip strength (HGS < 27 kg), reduced appendicular skeletal mass index (ASMI < 7 kg/m^2^), and reduced gait speed ≤ 0.8 m/s [40,46]. The prevalence of comorbidities was determined using the Charlson comorbidity index, as described previously by us [3].

### 4.2. Ethics Approval

The study participants were recruited from the Department of Neurology and Stroke Medicine at Rehman Medical Institute, Peshawar, Pakistan, after obtaining informed consent. An ethics approval was obtained from the Office of the Dean for the Human Research Ethics Committee of the Rehman Medical Institute (Reference number: HREC-24-05-03-02, dated: 5 March 2023). This study was conducted under the Declaration of Helsinki [47].

### 4.3. HGS and Body Composition

HGS was measured using a digital handgrip dynamometer (CAMRY, South El Monte, CA, USA), as described previously by us [48]. We used a bioelectrical impedance analysis scale (RENPHO, Dubai, UAE) to measure the body composition, including lean mass, fat content, phase angle, appendicular skeletal muscle mass (ASM), and appendicular skeletal muscle mass index (ASMI) [48].

### 4.4. Measurement of Physical Capacity

We used the short physical performance battery (SPPB) to measure the physical capacity of the study population. The battery is a composite test, including a 4 m walking test (4MWT), balance, and a five-times chair-stand (5-STS) test.

For the measurement of postural balance, participants were asked to perform three stances: side-by-side stand, semi-tandem stand, and tandem stand. In each stance, the participants were instructed to hold their position for 10 s. The side-by-side stand required participants to stand with their feet together. The semi-tandem stand involved placing the side of the heel of one foot touching the big toe of the other foot. The tandem stand required participants to place the heel of one foot directly in front of and touching the toes of the other foot. Timing began when the participant was in position and stopped after 10 s or when the participant lost balance.

A 6 m course was marked on a flat surface to perform the 4MWT. Participants were instructed to walk at their comfortable, self-selected pace. The first and last meters were used for acceleration and deceleration, respectively. The middle four meters were the timed section where gait speed was measured. Timing began when the participant’s first foot crossed the start line of the timed section and stopped when the first foot crossed the end line of the timed section. A handheld stopwatch was used for timing. Two trials were conducted, and the fastest time was recorded. Gait speed was calculated by dividing the distance (4 m) by the time taken to walk that distance, resulting in a meter per second (m/s) [49].

For the 5-STS test, participants were asked to sit on a chair with a backrest, stand up, and sit down five times as quickly as possible without using their arms. Timing began when the participant’s back left the backrest and stopped when they sat down after the fifth stand. The total time taken to complete the five repetitions was recorded [49].

Each of the three components of SPPB was assigned a score ranging from zero (worst score) to four (best score), and the total score of the three tests was used to measure the physical capacity of participants [50]. We used a digital pedometer to measure daily step count from the past four weeks, as described previously [51].

### 4.5. Measurement of Circulating Biomarkers

We used an ELISA kit to measure plasma zonulin levels (catalog number: K5601, Immundiagnostik AG, Bensheim, Germany), as described elsewhere [3]. We used ELISA kits to quantify 8–isoprostanes (Cayman Chemical, Ann Arbor, MI, USA), creatine kinase (CK), and C-reactive proteins (CRP) (R&D Systems, Minneapolis, MN, USA), as described elsewhere by us [51,52].

### 4.6. Statistical Analysis

The analysis of variance with Tukey’s post hoc test was used to compare groups. We used correlation analysis to measure the association between plasma zonulin and sarcopenia parameters. The differences between baseline and one year were measured using paired *t*-tests. Data are presented as mean and standard deviation, and the *p*-value < 0.05 was taken as statistically significant. Data were analyzed using GraphPad Prism 8 (Graphstats Technologies Private Limited, Bangalore 560035, India).

## Figures and Tables

**Table 1:** Baseline characteristics of controls, normotensive and hypertensive AD patients taking ACE inhibitors or other anti-hypertensive medications. * *p* < 0.05 vs. controls using one-way analysis of variance. # *p* < 0.05 vs. baseline for the same group using paired *t*-test, (*n* = 55–64 per group). (BMI; body mass index, ASM; appendicular skeletal mass, ACE; angiotensin converting enzymes, MMSE; mini-mental state examination).

	Controls (*n* = 64)	Normotensive (*n* = 61)		Hypertensive (*n* = 102)			
				ACE Inhibitors (*n* = 55)		Other Anti-Hypertensive (*n* = 57)	
		Baseline	One Year	Baseline	One Year	Baseline	One Year
Age (years)	74.6 ± 4.5	76.3 ± 3.8	77.3. ± 3.9	74.9 ± 4.2	75.9 ± 4.2	76.1 ± 4.7	77.1 ± 4.7
BMI (Kg/m^2^)	24.1 ± 2.4	24.6 ± 3.1	25.1 ± 3.8	25.4 ± 3.7	25.2 ± 3.9	26.4 ± 3.4	26.8 ± 3.4
ASM (Kg)	26.9 ± 3.6	23.5 ± 2.5 *	23.1 ± 2.8 *	24.4 ± 3.3	24.8 ± 3.8	24 ± 2.3	23.8 ± 2.6 *
Percent fat	28.4 ± 3.5	27.3 ± 4.2 *	27.8 ± 4.2	28.4 ± 3.7	27 ± 4.5 *	26.8 ± 3.5 *	27.3 ± 4.3
Phase angle	5.93 ± 0.8	5.73 ± 0.5 *	5.82 ± 0.5	5.71 ± 0.6 *	5.84 ± 0.6 #	5.68 ± 0.5 *	5.61 ± 0.6 *
Daily steps count	8271 ± 1483	4751 ± 1518 *	4162 ± 1041 *	5381 ± 1273 *	5841 ± 1501 *	4813 ± 953 *	4619 ± 1051 *
Creatine kinase (IU/L)	185.7 ± 37.8	252.6 ± 56.1 *	268.3 ± 42.6 *	247.2 ± 38.5 *	236.3 ± 48.2 *	229.6 ± 38.6 *	248.5 ± 39.5 *#
MMSE scores	26.4 ± 2.1	16.5 ± 2.6 *	16.9 ± 2.9 *	17.5 ± 3.1 *	16.2 ± 2.7 *	16.1 ± 2.6 *	15.6 ± 2.7 *
Charlson comorbidity index	0.68 ± 0.12	1.35 ± 0.43	1.38 ± 0.45	1.53 ± 0.38	1.5 ± 0.37	1.48 ± 0.4	1.5 ± 0.41
Sarcopenia cases, *n* (%)	13 (20.31)	16 (26.2)	18 (29.5)	15 (25.5)	15 (25.5)	18 (31.7)	19 (33.3)

**Table 2:** Linear regression analysis showing the coefficients of determination of plasma zonulin levels with MMSE, HGS, ASMI, gait speed, cumulative SPPB, CK, CRP, and 8-isoprostanes in controls, normotensive, and hypertensive AD patients taking ACE inhibitors or other anti-hypertensive medications. * *p* < 0.05. MMSE; mini-mental state examination, HGS; handgrip strength, ASMI; appendicular skeletal mass index, SPPB; Short physical performance battery, CK; creatine kinase, CRP; c-reactive proteins, ACE; angiotensin-converting enzymes.

	Controls (*n* = 64)	Normotensive (*n* = 61)		Hypertensive (*n* = 102)			
				ACE Inhibitors (*n* = 55)		Other Anti-Hypertensive (*n* = 57)	
		Baseline	One Year	Baseline	One Year	Baseline	One Year
MMSE	0.019	0.041	0.028	0.071	0.123 *	0.057	0.036
HGS	0.096 *	0.173 *	0.127 *	0.214 *	0.149 *	0.107	0.083
ASMI	0.017	0.008	0.029	0.128 *	0.094	0.025	0.024
Gait speed	0.107 *	0.097	0.082	0.187 *	0.119 *	0.11 *	0.063
Cumulative SPPB	0.069	0.079	0.061	0.155 *	0.133 *	0.077	0.112 *
CK	0.106 *	0.127 *	0.053	0.097	0.176 *	0.041	0.056
CRP	0.086	0.198 *	0.217 *	0.201 *	0.179 *	0.153 *	0.185 *
8-isoprostanes	0.063	0.084	0.091	0.151 *	0.183 *	0.078	0.049

## Data Availability

The original contributions presented in this study are included in the article. Further inquiries can be directed to the corresponding author.
